# Active control of excitonic strong coupling and electroluminescence in electrically driven plasmonic nanocavities

**DOI:** 10.1126/sciadv.adt9808

**Published:** 2025-05-28

**Authors:** Junsheng Zheng, Alexey V. Krasavin, Ruoxue Yang, Zhenxin Wang, Yuanjia Feng, Longhua Tang, Linjun Li, Xin Guo, Daoxin Dai, Anatoly V. Zayats, Limin Tong, Pan Wang

**Affiliations:** ^1^ Interdisciplinary Center for Quantum Information, New Cornerstone Science Laboratory, State Key Laboratory of Extreme Photonics and Instrumentation, College of Optical Science and Engineering, Zhejiang University, Hangzhou 310027, China.; ^2^ Department of Physics and London Centre for Nanotechnology, King’s College London, Strand, London WC2R 2LS, UK.; ^3^ Jiaxing Key Laboratory of Photonic Sensing & Intelligent Imaging, Intelligent Optics & Photonics Research Center, Jiaxing Research Institute Zhejiang University, Jiaxing 314000, China.; ^4^ Collaborative Innovation Center of Extreme Optics, Shanxi University, Taiyuan 030006, China.

## Abstract

Enhancement and active control of light-matter interactions at the atomic scale is important for developing next-generation nanophotonic and quantum optical devices. Here, we demonstrate electric control of excitonic strong coupling and electroluminescence (EL) by integrating a semiconductor monolayer into a nanometer gap of single electrically driven nanocube-on-mirror plasmonic nanocavities, which provide unmatched optical and electrical confinement. In particular, in a strongly coupled system of nanocavity plasmons and tungsten diselenide (WSe_2_) excitons, an ultrastrong electric field generated in the nanocavity gap enables reversible modulation of the Rabi splitting between ~108 and 102 milli–electron volts with a bias of only 2.5 volts. In the quantum tunneling regime (realized by decreasing the gap size), by injection of carriers into a nanocavity-integrated tungsten disulfide (WS_2_) monolayer, spectrally tunable EL (controlled by the bias polarity) is achieved with a room-temperature quantum efficiency reaching ~3.5%, showing an improvement of more than 10^3^ times over previous works.

## Teaser

Active control of atomic-scale light-matter interactions is demonstrated in electrically driven functional plasmonic nanocavities.

## INTRODUCTION

The study of interactions between light and matter plays a pivotal role in the advance of optical science and technologies. Squeezing electromagnetic fields down to a deep subwavelength scale with plasmonic or dielectric nanostructures enables access to the extreme light-matter interactions at the atomic scale (*1*–*7*). This is of great interest for both state-of-the-art fundamental research and development of high-performance nanophotonic and quantum optical devices ranging from nanoscale light sources and optical modulators to photodetectors. In this context, atomic-scale light-matter interactions between solid-state emitters and photonic nanostructures with high local density of optical states have recently attracted considerable research interest (*5*, *6*). In such coupled systems, in addition to a weak coupling regime when the emitters exhibit Purcell-enhanced spontaneous emission (*8*–*10*), strong coupling can be achieved to form hybridized light-matter states when the rate of energy exchange between light and matter exceeds the dissipation rates in the system (*11*–*14*). For further fundamental exploration and practical applications, it is of immense importance to add another degree of freedom and actively control atomic-scale light-matter interactions represented by strong coupling and spontaneous emission, especially in a straightforward electric way. This promises to uncover new physical phenomena via the rich interactions between optical and electric effects in functional materials and leads to a variety of applications in nanoscale optoelectronics and quantum optical technologies.

**Fig. 1.**
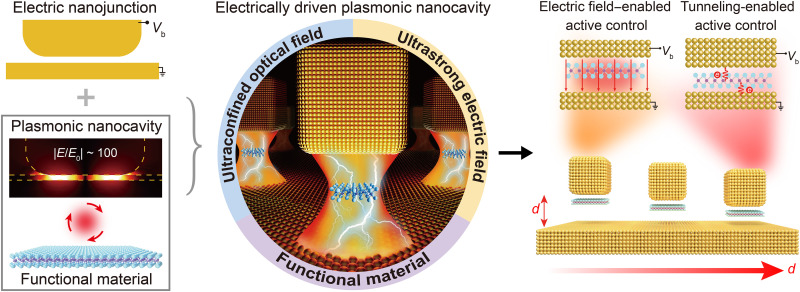
Conceptual illustration of active control of light-matter interactions in electrically driven functional plasmonic nanocavities. The interplay between ultraconfined optical field, ultrastrong electric field, and functional material in a nanoscale spatial region of an electrically driven plasmonic nanocavity enables active control of atomic-scale light-matter interactions, driven, depending on the gap size, by strong-field or quantum tunneling effects.

In contrast to dielectric photonic nanostructures, their metallic plasmonic counterparts can simultaneously perform both optical and electric functions. Thus, they have long been envisioned to have the ability to merge photonics and electronics at the nanoscale (*15*, *16*), which opens up vast opportunities for robust electric control of atomic-scale light-matter interactions. Among various plasmonic nanostructures, plasmonic nanocavities, which are produced by separating two metallic objects with a nanometer-thick dielectric gap (e.g., nanoparticle-on-mirror nanocavities and metallic nanoparticle dimers), have recently attracted increasing research interest. In particular, they provide extremely confined optical fields in the gap for enhancing light-matter interactions at the atomic scale (*17*, *18*) and manifest fascinating quantum mechanical effects (*19*–*21*). Moreover, upon application of a bias voltage of only several volts across the nanometer-thick gap, they offer an attractive means to achieve ultrastrong electric fields up to the level of volts per nanometer, additionally enabling engineered electron tunneling. Therefore, it is of great interest to merge the unique properties of an optical nanocavity and an electric nanojunction to realize electrically driven functional plasmonic nanocavities (as schematically shown in Fig. 1). This enables strong interplay between ultraconfined optical field, ultrastrong electric field, quantum tunneling effect, and functional material in a nanoscale spatial region and provides unprecedented opportunities for active control of atomic-scale light-matter interactions.

Here, by integrating a semiconductor monolayer into the nanometer gap of electrically driven single-crystal nanocube-on-mirror (NCoM) plasmonic nanocavities, we demonstrate robust electric control of excitonic strong coupling and electroluminescence (EL). In particular, upon functionalization of the nanocavity gap with a WSe_2_ monolayer, strong coupling between nanocavity plasmons and WSe_2_ excitons is achieved. Using an ultrastrong electric field (~0.5 V/nm) produced in the gap with a bias of only 2.5 V, the Rabi splitting can be reversibly modulated between ~108 and 102 meV through the control of the number of involved excitons. With the further decrease in the gap size to enter the quantum tunneling regime, electric excitation of excitonic luminescence from a nanocavity-integrated WS_2_ monolayer with an external quantum efficiency (EQE) as high as 3.5% is demonstrated by injecting carriers into the monolayer via tunneling. The spectral characteristics of the EL have been engineered via selective generation of charged or neutral excitons by controlling the bias polarity.

## RESULTS

### Fabrication of electrically driven plasmonic nanocavities

**Fig. 2.**
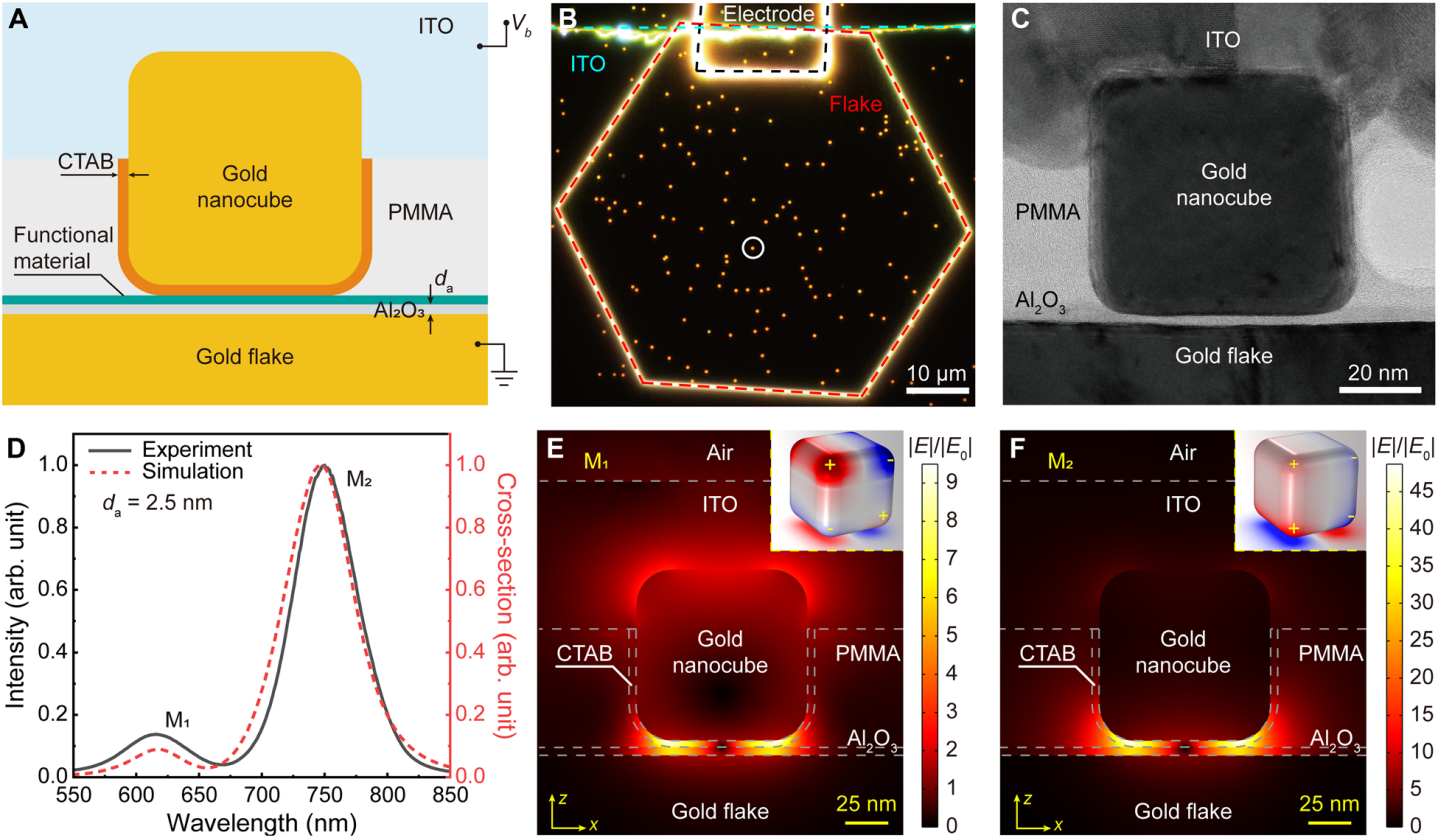
Fabrication and characterization of electrically driven plasmonic nanocavities. (**A**) Schematic illustration of an electrically driven functional plasmonic nanocavity, formed by an NCoM structure (integrated with a functional material) with a transparent conductive layer of ITO at the top, simultaneously providing optical and electric access. The size of the nanocavity gap is controlled by precisely tuning the thickness of the alumina layer (*d*_a_). (**B**) Dark-field scattering image of an ensemble of electrically driven bare plasmonic nanocavities formed on a 50-nm–thick gold flake with *d*_a_ of 2.5 nm. The black, red, and cyan dashed lines indicate the outlines of the gold electrode, the gold flake, and the ITO layer, respectively. (**C**) Cross-sectional TEM image of an electrically driven bare plasmonic nanocavity. (**D**) Experimentally measured (solid line) and numerically calculated (dashed line) scattering spectra of the electrically driven plasmonic nanocavity indicated by a white circle in (B). arb. unit, arbitrary unit. (**E** and **F**) Numerically simulated near-field distribution of the electric field in the *xz* plane corresponding to nanocavity modes M_1_ (E) and M_2_ (F) marked in (D), normalized to the magnitude of the incident field *E*_0_. Insets: The corresponding charge density distributions on the surfaces of the gold nanocube and flake.

Figure 2A shows a schematic illustration of an electrically driven functional plasmonic nanocavity. The nanocavity is formed by depositing a gold nanocube, which is capped with a bilayer of cetyltrimethylammonium bromide (CTAB), on the top of a gold mirror (also working as a bottom electrode) covered by an alumina spacer and a layer of functional material (e.g., a semiconductor monolayer in this work). For electric integration, the gold nanocube is partially embedded in an insulating poly(methyl methacrylate) (PMMA) layer and then coated with a transparent conductive layer of indium tin oxide (ITO), acting as a top electrode. When a bias is applied between the top and the bottom electrodes, a strong electric field is generated in the nanocavity gap, while, at the same time, the nanocavity can still be optically accessed through the transparent ITO layer to produce extremely confined electromagnetic fields in the same gap.

In practice, it is very challenging to fabricate electrically driven plasmonic nanocavities using deposited metal films as the mirror due to their granular polycrystalline structure. These films not only introduce a substantial optical loss that can degrade the strength of light-matter interactions (*22*, *23*) but also make the nanocavity easily damaged or broken by surface roughness–induced protrusions in the nanocavity gap under a strong electric field. In this work, chemically synthesized gold nanocubes (with an average size of 60 nm and a CTAB thickness of ~1.8 nm; see the Materials and Methods and fig. S1 for details) and gold flakes (fig. S2) (*7*, *22*), having both a single-crystal structure and an ultrasmooth surface, were used as building blocks for the formation of electrically driven plasmonic nanocavities with low optical loss and high-stability electric nanojunctions. The procedure for the fabrication of electrically driven functional plasmonic nanocavities is explained in Materials and Methods (see also figs. S3 and S4). Electrically driven bare (not functionalized) plasmonic nanocavities were first constructed on a gold flake (see fig. S5 for the scanning electron microscopy image) to investigate their optical properties. Benefitting from high transparency of the ITO layer, nanophotonic modes of the electrically driven bare plasmonic nanocavities can be optically excited and observed as yellow spots in a dark-field scattering image (Fig. 2B and see fig. S6 for the experimental setup). A cross-sectional transmission electron microscopy (TEM) image of an electrically driven bare plasmonic nanocavity (Fig. 2C and see the Materials and Methods for details) shows that the gold nanocube is perfectly separated by a dielectric spacer (produced by the 1.8-nm–thick CTAB and 2.5-nm–thick alumina layers) from the gold flake forming a uniform nanocavity gap with ultrasmooth interfaces and is also electrically contacted with the top ITO layer. The measured scattering spectrum (Fig. 2D, solid line) reveals two main scattering peaks located at 615 and 751 nm (labeled as modes M_1_ and M_2_, respectively), which match very well with the optical modes in a numerically simulated scattering spectrum (Fig. 2D, dashed line; figs. S7 and S8; and see Materials and Methods for simulation details) and correspond to the excitation of flake-coupled quadrupolar (Fig. 2E) and transversal dipolar (Fig. 2F) modes of the nanocube (*22*, *23*), respectively. The simulations show that greatly increased local fields can be achieved in the nanometer-thick dielectric gap, which is important for the enhancement of light-matter interactions at the atomic scale. By controlling the thickness of the dielectric gap, the resonance peaks of these plasmonic modes can be precisely tuned to the desired wavelengths (fig. S9).

### Active modulation of strong plasmon-exciton coupling

**Fig. 3.**
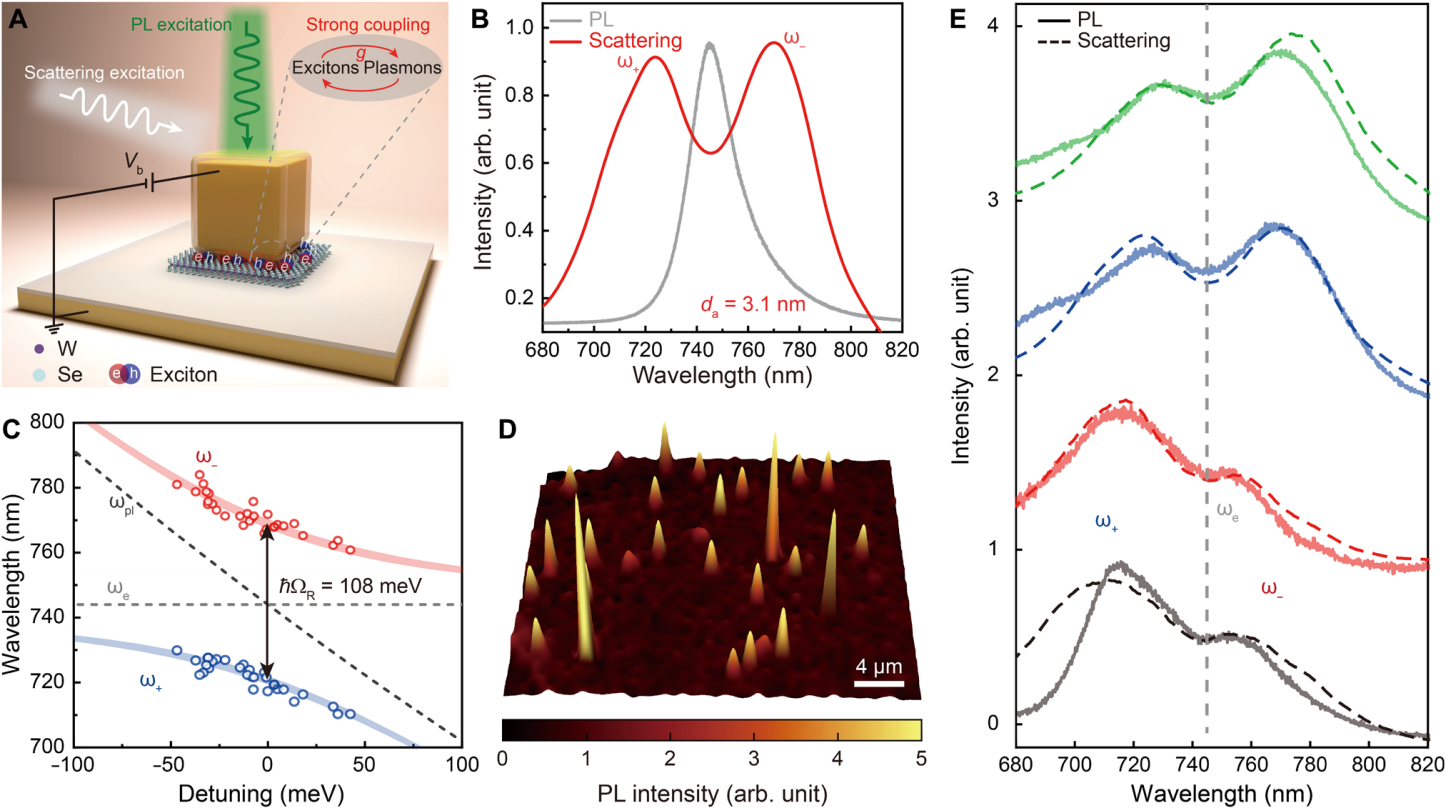
Strong plasmon-exciton coupling in unbiased single electrically driven functional plasmonic nanocavities. (**A**) Schematic illustration of the device. The white and green incident beams indicate the light used for scattering and PL measurement, respectively. (**B**) Measured scattering spectrum of an unbiased electrically driven plasmonic nanocavity (red solid line), which is functionalized with a WSe_2_ monolayer in the gap (*d*_a_ = 3.1 nm). For reference, a PL spectrum of a WSe_2_ monolayer on a sapphire substrate (gray solid line) is also provided. (**C**) Measured spectral positions (hollow circles) of hybridized plasmon-exciton states as a function of the detuning between the energies of the initial plasmonic mode (black dashed line) and the excitons (gray dashed line). The colored lines represent analytically calculated spectral dispersion of the hybridized modes with a Rabi splitting of 108 meV. (**D**) PL mapping of an ensemble of WSe_2_ monolayer–functionalized plasmonic nanocavities under the excitation with a 532-nm laser light. (**E**) Measured PL (solid lines) spectra for nanocavities with various detuning between the energies of the initial plasmonic modes and excitons. The corresponding dark-field scattering spectra [dashed lines; selected from spectra used to plot the map in (C)] are presented for reference.

In addition to the extreme light confinement in the nanocavity (*17*, *18*), it is very straightforward to achieve an ultrastrong electric field inside the nanocavity gap upon application of a bias between the gold nanocube and the flake, which opens opportunities for electric tuning of the properties of functional materials integrated in the gap and subsequently for active modulation of atomic-scale light-matter interactions. Here, we exploit these advantages for electric modulation of strong plasmon-exciton coupling (as schematically shown in Fig. 3A), which is highly required for practical applications in optoelectronics and quantum information processing (*24*, *25*), but challenging to realize because of the limited tunability of excitons in functional materials.

For the experimental demonstration, a monolayer of WSe_2_, which hosts in-plane oriented A excitons at room temperature with an emission peak around 1.67 eV (*26*), was integrated into the nanocavity gap to form electrically driven functional plasmonic nanocavities (Fig. 3A) via transfer printing (figs. S3 and S4 and see the Materials and Methods for experimental details). It is important that the WSe_2_ monolayer outside the nanocavities was removed using oxygen plasma etching (fig. S10) to eliminate its influence on the measurement of strong coupling in scattering and photoluminescence (PL) spectra. When the resonance peak of mode M_2_ is matched to the emission peak of the A excitons (gray solid line in Fig. 3B) by adjusting the alumina layer thickness *d*_a_ to ~3.1 nm, a typical mode splitting profile arising from strong coupling can be clearly observed in the nanocavity scattering spectrum (red solid line in Fig. 3B), presenting two hybridized plasmon-exciton modes. The excitons in the monolayer are in-plane in their nature and thus are coupled to the in-plane components of the electric field shown in figs. S11 and S12. Figure 3C further presents the evolution of the peak wavelengths of the two plasmon-exciton branches (upper branch ω_−_ and lower branch ω_+_, which are obtained by fitting the scattering spectra with two Lorentzian line shapes; fig. S13) (*27*) with controlled detuning ($\delta =\omega_{\text{pl}}-\omega_{e}$) between the plasmon and exciton energies. Here, the detuning was varied using WSe_2_-functionalized nanocavities with various combinations of the alumina thickness and nanocube size. The evolution exhibits a distinct anticrossing behavior with Rabi splitting $\hbar \Omega_{R}$ = 2ℏg = 108 meV (where g represents the coupling strength). This value satisfies the criteria for strong coupling $\Omega_{R}\geq |\left(\kappa +\gamma \right)/2|$ (*28*), where the excitonic linewidth (ℏκ) is ~57 meV and the average plasmon linewidth (ℏγ) for mode M_2_ is ~100 meV. This observation is further confirmed using Tavis-Cummings model (*29*) (see the Materials and Methods for details) to describe the strong coupling effect, which can reproduce the experimental results very well (red and blue curves in Fig. 3C). To further confirm the strong coupling between nanocavity plasmons and WSe_2_ excitons, we also measured PL from single electrically driven functional plasmonic nanocavities. Under the excitation with a 532-nm laser, PL signals from single nanocavities can be observed with almost zero emission background from regions outside the nanocavities (Fig. 3D). As presented in Fig. 3E, clear energy splitting profiles can be observed in the PL spectra from nanocavities with various detuning between the energies of the plasmonic modes and excitons (solid lines), showing a good agreement with the corresponding scattering spectra (dashed lines). All these results convincingly demonstrate that the electrically driven functional plasmonic nanocavities reach the strong coupling regime.

**Fig. 4.**
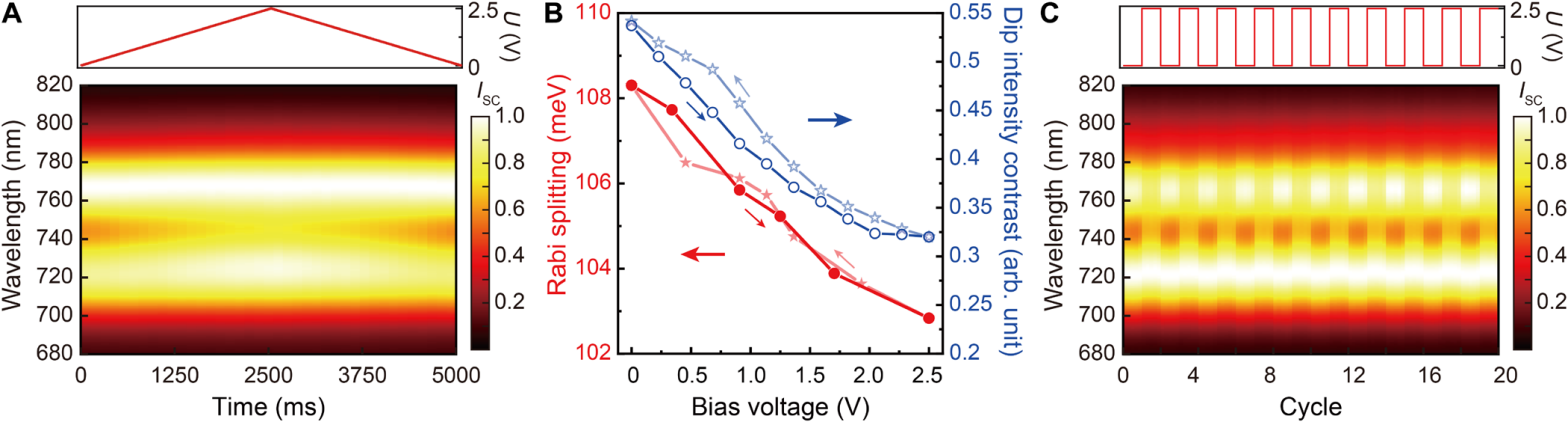
Electric modulation of strong plasmon-exciton coupling. (**A**) Spectral map showing the evolution of the scattering spectra of an electrically driven functional plasmonic nanocavity with a continuously increasing and then a reversely decreasing bias as indicated in the top panel. The color represents the normalized scattering intensity. (**B**) Rabi splitting (red solid circles and stars) and dip intensity contrast (blue hollow circles and stars) extracted from the scattering spectra with forward and backward (indicated by downward and upward arrows, respectively) scanning of the bias during the modulation process presented in (A). (**C**) Spectral map showing the evolution of the scattering spectra with alternative switching of the bias between 0 and 2.5 V (as indicated in the top panel). The color represents the normalized scattering intensity.

Upon application of a bias between the top ITO layer and the bottom gold flake, electric modulation of the strong plasmon-exciton coupling was further realized (Fig. 4A). With a gradual increase in the bias from 0 to 2.5 V (corresponding to the nanocavity electric field of ~0.5 V/nm), the Rabi splitting in the scattering spectrum gradually decreases, which is accompanied by a clear decrease in the intensity contrast of the scattering dip [defined as $2\left(I_{\text{peak}}-I_{\text{dip}}\right)/\left(I_{\text{peak}}+I_{\text{dip}}\right)$, where $I_{\text{peak}}$ and $\;I_{\text{dip}}$ are the peak and dip intensity values in the normalized scattering spectra, respectively]. When the applied bias is lifted, the strong plasmon-exciton coupling in the WSe_2_-functionalized nanocavity recovers back to its initial state. The modulation of the Rabi splitting (between ~108 and 102 meV) and the corresponding dip intensity contrast (between ~0.54 and 0.32) with the variation of the bias can be explicitly seen in Fig. 4B. There is a small hysteresis in the modulation dependence, which can be caused by the temporal trapping of the dissociated carriers by atomic defects in the WSe_2_ monolayer (*30*) and the transfer printing–introduced ionic contamination on its surface (*31*), slowing down the system response to the electric field. From a theoretical point of view, the Rabi splitting $\Omega_{R}$ is proportional to the square root of the number of excitons ($\sqrt{N}$) involved in the strong coupling and inversely proportional to the square root of the volume ($\sqrt{V}$) of the plasmonic mode, as $\Omega_{R}=\mu_{m}\sqrt{4\hbar \mathrm{Nc}/\left(\lambda \varepsilon \varepsilon_{0}V\right)}$ (where λ and $\mu_{m}$ are the transition wavelength and the dipole moment of the excitons, respectively, ε is the relative permittivity of the material surrounding the semiconductor monolayer, ℏ is the reduced Planck constant, and $\varepsilon_{0}$ is the free-space permittivity) (*11*, *32*). Therefore, in the case of an unchanged mode volume, the electric modulation of the strong plasmon-exciton coupling in the WSe_2_-functionalized nanocavity is mainly due to the variation of the number of the involved excitons, which can be estimated to decrease from ~100 to 90 with the increase of the bias from 0 to 2.5 V (fig. S14 and see the Materials and Methods for details). This can be understood from the fact that the stability of excitons can be greatly reduced with the application of a strong static electric field in the out-of-plane direction, which eventually leads to their dissociation and a decrease in the density of excitons in the WSe_2_ monolayer (*33*, *34*). For an electrically driven bare plasmonic nanocavity without a WSe_2_ monolayer functionalization in the gap, its scattering spectrum is unchanged under the scanning of the applied bias (fig. S15), which excludes a contribution from electric field–induced variation of the nanocavity structure to the observed modulation effect. Figure 4C presents a map showing the evolution of the scattering spectra from the electrically driven functional nanocavity, with the bias alternatively switched between 0 and 2.5 V, which illustrates the excellent repeatability in the electric modulation of the strongly coupled system.

Compared to existing active modulation approaches, such as electrostatic gating (*35*–*37*), thermal tuning (*36*, *38*), and electrochemical switching (*39*), the electric field–based modulation approach demonstrated here provides advantages including a much lower operation bias of only 2.5 V, a higher modulation bandwidth [theoretically limited only by the estimated device resistance-capacitance (*RC*) time constant of ~240 fs, as discussed in fig. S16, therefore potentially reaching the terahertz level] and an all-solid-state architecture. Moreover, different from the electrostatic gating approach that only works for conductive excitonic materials with at least a micrometer-scale lateral size (necessary for an electric contact) (*35*–*37*), the electric field–based modulation approach realized here can be readily applied to strong coupling systems using excitonic materials such as molecules or quantum dots. Therefore, it represents a universal means for strong coupling modulation, which is attractive not only for electric control of optical signals at the nanoscale but also for applications such as quantum manipulation and quantum information processing.

### Spectrally tunable excitonic EL

**Fig. 5.**
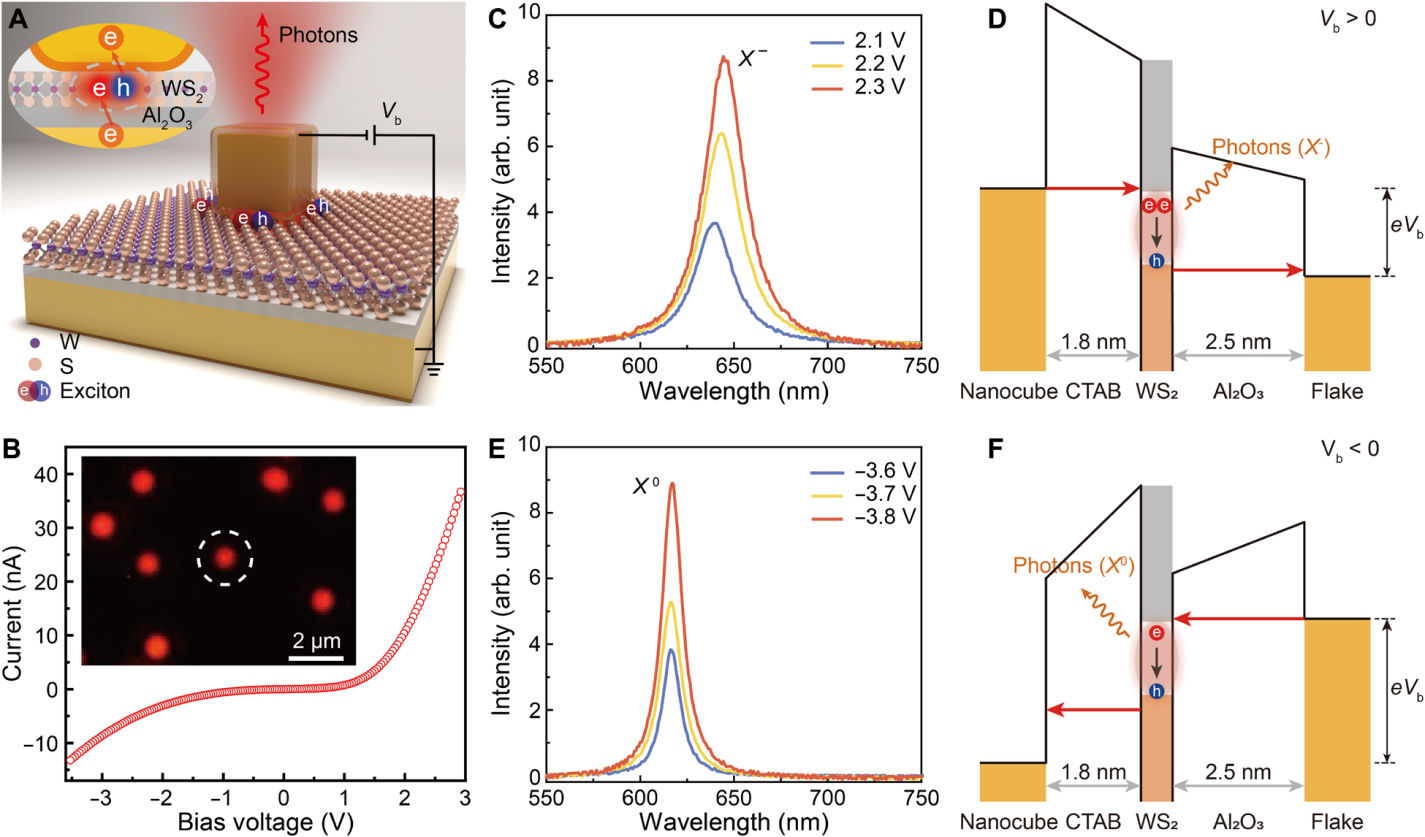
Electric excitation and control of excitonic luminescence by quantum tunneling. (**A**) Schematic illustration of a quantum tunneling–enabled excitonic luminescence process in an electrically driven plasmonic nanocavity functionalized with a WS_2_ monolayer. (**B**) Current-voltage characteristic of a tunneling device containing ~50 plasmonic nanocavities functionalized with a WS_2_ monolayer (*d*_a_ = 2.5 nm). Inset: Detected red light emission [captured with a color charge-coupled device (CCD) camera] from nanocavities under a forward bias of 2.3 V. (**C** and **E**) Measured emission spectra from the nanocavity marked in (B) under various forward (C) and backward (E) biases. (**D** and **F**) Energy diagrams of the electrically driven functional plasmonic nanocavity under forward (D) and backward (E) biases.

With a further decrease in the nanocavity gap size, it is possible to activate the quantum tunneling regime, which can be exploited for direct electric excitation of luminescence from nanocavity-integrated active materials via the injection of electrons and holes into them, which is followed by electron-hole recombination. Moreover, the plasmonic nanocavities featuring large Purcell factors provide an unprecedented ability for the engineering and enhancement of the EL. Therefore, the architecture developed here also presents great interest for the realization of electrically driven nanoscale light sources with high quantum efficiency. To demonstrate this, we integrated a monolayer of WS_2_, which is a two-dimensional n-type semiconductor (*40*, *41*) offering stronger PL (compared to WSe_2_) dominated by negatively charged excitons (fig. S17), into electrically driven plasmonic nanocavities having an alumina spacer with a decreased thickness (*d*_a_ = 2.5 nm). In this case, the electron tunneling becomes pronounced, leading to the onset of the carrier injection–driven excitonic luminescence from the WS_2_ monolayer, which is emitted to the far field via the excitation of nanocavity plasmons (Fig. 5A). The key difference to the strong coupling experiments discussed above is that the WS_2_ monolayer has a considerably blue-shifted excitonic frequency compared to that in the WSe_2_ monolayer, which, together with the change of the gap size, leads to the situation when the spectral positions of the plasmonic modes do not match the excitonic frequency (fig. S18). Thus, the system experiences a transition from the strong coupling regime to the weak coupling regime and demonstrates Purcell-enhanced EL.

A characteristic current-voltage curve for a tunneling device containing ~50 nanocavities is presented in Fig. 5B. The tunneling current is much higher than that in the strong coupling device (fig. S19; particularly, ~0.45 nA versus 0.03 nA for single nanocavities under a forward bias of 2.5 V). With the increase in the bias voltage, the current shows a superlinear increase, which confirms the tunneling nature of the electronic transport through the gap of the plasmonic nanocavities. Under an application of a forward bias of 2.3 V, spots of red-color light emission (inset of Fig. 5B; see the Materials and Methods and fig. S20 for experimental details), each corresponding to the position of an individual electrically driven functional nanocavity, were observed. Figure 5C further presents the emission spectra of a single nanocavity (encircled in Fig. 5B) measured under a varied forward bias, which exhibit an emission peak around 643 nm having a linewidth of ~25 nm. The observed behavior agrees well with the PL characteristics of WS_2_ monolayers at room temperature (fig. S17), indicating that the EL originates from the radiative recombination of charged (*X*^−^) excitons in the integrated WS_2_ monolayer, which are created by the quantum tunneling (*42*–*45*). In particular, as schematically presented in Fig. 5D, under a sufficiently high forward bias (when the applied voltage is high enough to the presence of empty states in the receiving materials for both junctions), electrons of the gold nanocube tunnel into the conduction band of the integrated WS_2_ monolayer, while, at the same time, electrons in the valance band of the WS_2_ monolayer tunnel to the gold flake, generating holes in their original places, thus allowing the formation of excitons in the WS_2_ monolayer. Because of the differences in the thicknesses of the CTAB (~1.8 nm) and alumina (~2.5 nm) layers and the corresponding barrier heights, electrons in the conduction band of the n-type WS_2_ monolayer accumulate more efficiently than holes in the valence band, resulting in the generation of negatively charged excitons that emit light with a wavelength around 643 nm at room temperature (*46*, *47*). The intensity of the excitonic EL rises with the increase in the applied bias. Although the electric field created in the gap leads to the dissociation of the excitons as explained above, it is vastly overcompensated by the exponential increase in the tunneling current generating them, leading to the increase in the EL signal. When the applied bias is less than ~2.0 V (not high enough to generate excitons in the semiconductor monolayer), light emission can also be obtained from the WS_2_-functionalized electrically driven plasmonic nanocavities due to an inelastic electron tunneling process (*48*). However, the resulting emission spectrum has a peak wavelength of ~840 nm and a broad linewidth of ~122 nm (fig. S21). With the gradual increase in the applied bias from 1.8 to 2.1 V, the transition in the emission spectrum from inelastic tunneling excited luminescence to excitonic EL can be observed.

Light emission was also observed from the same nanocavity when a backward bias was applied. However, the emission spectra show a blue-shifted peak wavelength of ~617 nm and a narrower linewidth of ~13 nm (Fig. 5E), which is totally different from the emission characteristic of the charged excitons in WS_2_ monolayers and can be assigned to the radiative recombination of their neutral (*X*^0^) A-type counterparts (*41*, *46*). In contrast to the forward-biased case, under a backward bias, holes in the valence band of the n-type WS_2_ monolayer accumulate more efficiently than electrons in the conduction band (Fig. 5F), which results in compensation of the natural n-doping of WS_2_ and generation of *X*^0^ excitons that emit light around 617 nm observed in the experiment. Therefore, the difference in the thicknesses of the dielectric barriers surrounding the WS_2_ monolayer plays an important role in the active control of the EL, which is difficult to realize with other electric excitation approaches. The EQE (the ratio between emitted photons and tunneled electrons) of the electrically driven nanocavity under a forward bias of 2.3 V is estimated to be ~3.5% at room temperature (see the Materials and Methods for details). It is more than 10^3^ times higher than EQEs of tunneling-excited EL of semiconductor monolayers demonstrated previously (*44*, *45*, *49*–*52*), due to the Purcell enhancement provided by plasmonic mode M_1_ of the low-loss single-crystal nanocavity. The EQE can be further improved by optimizing the structural parameters of the nanocavities to realize a better match between the excitonic emission spectrum and the resonant wavelength of the plasmonic mode (fig. S22).

EL has been also observed from nanocavities functionalized with other semiconductor monolayers, such as WSe_2_ (fig. S23). The EQE under a forward bias of 3.2 V is estimated to be ~7.5%, which is higher than that for WS_2_ due to a better match of the excitonic emission wavelength with the spectral position of the dipolar plasmonic mode M_2_. As a result, the linewidth of its EL spectrum is considerably broader than that of the PL spectrum (54 nm versus 26 nm, respectively; compare Fig. 3B and fig. S23) due to the Purcell effect. As the gap size is changed in comparison with the previous strong coupling experiment, the spectral positions of the plasmonic modes do not match the excitonic resonances, and the system is in a weak coupling regime, showing Purcell-enhanced EL (fig. S24). The spectrally tunable excitonic luminescence from semiconductor monolayers facilitated by tunneling is promising for the realization of electrically driven nanoscale light sources highly required for integrated electronic/photonic systems.

### Robustness of electrically driven plasmonic nanocavities

**Fig. 6.**
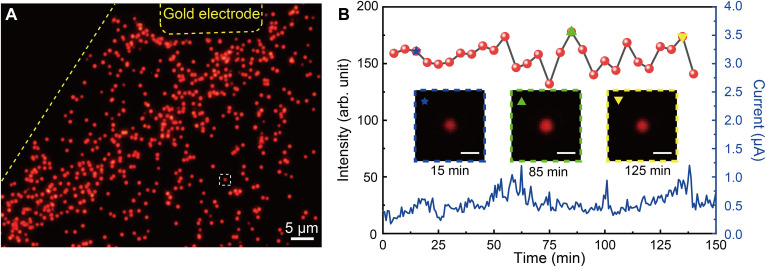
High robustness of electrically driven plasmonic nanocavities. (**A**) Image of light emission from hundreds of WS_2_-functionalized plasmonic nanocavities excited by quantum tunneling under a forward bias of 2.3 V. The yellow dashed lines indicate the outlines of the gold electrode and flake. (**B**) Time evolution of the detected integral light emission intensity (red solid dots) from the plasmonic nanocavity marked in (A) under a continuously applied forward bias of 2.3 V. The corresponding time-dependent tunneling current of the device containing ~1000 nanocavities is also plotted (blue line). Insets: Images of light emission from the selected nanocavity taken at 15, 85, and 125 min.

The robustness of electrically driven plasmonic nanocavities is critical for practical applications. In general, it is challenging to fabricate nanoscale electric junctions with a high yield and operation stability due to the facile breakdown of nanometer gaps under an ultrastrong electric field, caused by surface roughness of the metallic interfaces and consequent nonuniformity in the thickness of the dielectric spacer. The high crystal quality and ultrasmooth interfaces, together with the precise alumina deposition approach, enable rigorous fabrication of nanoscale electric structures with a high yield and robustness. Taking the EL as an example, hundreds of WS_2_-functionalized plasmonic nanocavities formed on a gold flake (over an area of ~2100 μm^2^) can be simultaneously lighten up with a uniform distribution of the emission intensities across the ensemble (Fig. 6A and fig. S25), indicating the exceptional fabrication yield reaching almost 100%. Furthermore, benefitting from the excellent structural quality, the electrically driven nanocavities can continuously work for a long time without a visible decrease in the emission intensity, as demonstrated by 2.5-hour measurements presented in Fig. 6B (the time-dependent tunneling current of the device is also provided for reference), which indicates their excellent long-term working stability and robustness.

## DISCUSSION

Using electric integration of single-crystal plasmonic nanocavities to simultaneously obtain ultraconfined optical field and ultrastrong electric field in the nanometer gap, we have demonstrated active control of atomic-scale light-matter interactions including electric modulation of strong plasmon-exciton coupling and spectrally tunable excitonic EL enabled by quantum tunneling. This approach can be generalized for electric control of other types of light-matter interactions such as nonlinear optical phenomena. In addition to semiconductor monolayers, other functional materials, such as dye molecules and quantum dots, can also be readily integrated into the nanocavity gap. Reciprocally, ultrafast optical control and generation of electric signals can also be possible with the electrically driven plasmonic nanocavities (*53*–*55*), which is of interest for applications in ultrafast photodetection and light-wave electronics. In conclusion, electrically driven plasmonic nanocavities provide a versatile platform for strong enhancement and active control of atomic-scale light-matter interactions and open opportunities for developing next-generation optoelectronic and quantum optical devices that can seamlessly interface electronics and photonics at the nanoscale.

## MATERIALS AND METHODS

### Synthesis of gold nanocubes

Single-crystal gold nanocubes with an average size of 60 nm were synthesized using a successive, seed-mediated method (*56*). First, to prepare initial CTAB-capped gold seeds, a freshly made ice-cold NaBH_4_ solution (0.6 ml, 0.01 M) was added into a mixed aqueous solution containing CTAB (9.75 ml, 0.1 M) and HAuCl_4_ (0.25 ml, 0.01 M). The resulting solution was rapidly stirred for 3 min and stored undisturbed for 3 hours before use. Second, gold nanospheres with an average diameter of ~10 nm were prepared by one-shot injection of an aqueous HAuCl_4_ solution (2 ml, 0.5 mM) into a mixed aqueous solution of cetyltrimethylammonium chloride (CTAC; 2 ml, 0.2 M), ascorbic acid (1.5 ml, 0.1 M), and the prepared CTAB-capped gold seeds (50 μl). The reaction was allowed to continue for 15 min. Then, the produced 10-nm gold nanospheres were collected by centrifugation and redispersed in 0.7 ml of an aqueous CTAC solution (0.02 M). Last, gold nanocubes with an average size of ~60 nm were prepared by one-shot injection of an aqueous HAuCl_4_ solution (6 ml, 0.5 mM) into a mixed aqueous solution of CTAC (6 ml, 0.1 M), ascorbic acid (0.39 ml, 0.01 M), NaBr (0.03 ml, 0.02 M), and the obtained solution with 10-nm gold nanospheres (6 μl). The resulting solution was stirred for 25 min and then treated with a centrifugation-driven depletion-induced flocculation procedure to selectively refine the nanocubes, thus obtaining high-quality gold nanocubes with a narrow size distribution.

### Fabrication of electrically driven functional plasmonic nanocavities

The procedure for the fabrication of electrically driven plasmonic nanocavities is schematically shown in fig. S3. First, a single-crystal gold flake with a thickness of 50 nm (synthesized on a glass slide) was transferred onto a silicon substrate (covered with a 300-nm–thick SiO_2_ layer) (*22*) to work as the gold mirror and bottom electrode for electrically driven plasmonic nanocavities (fig. S3A). Second, a gold strip electrode with a width of 20 μm was fabricated at the edge of the gold flake for electric connection. This was done by direct laser writing, followed by deposition of a Cr/Au (5/50 nm) layer and a lift-off (fig. S3B). Third, a nanometer-thick alumina layer was deposited on the surface of the gold flake using a molecular-assisted atomic layer deposition (ALD) approach to work as a dielectric spacer (fig. S3C) (*22*). This is critical for obtaining an alumina layer with a uniform thickness, which can be tuned by controlling the ALD reaction cycles. Fourth, functional material such as a WSe_2_ or a WS_2_ monolayer (grown on a sapphire substrate and purchased from SixCarbon Technology, China) was integrated into the nanocavities (fig. S3D) using a polydimethylsiloxane (PDMS)–assisted transfer method (fig. S4) (*7*). Fifth, a diluted solution of gold nanocubes was drop-casted onto the gold flake covered with an alumina layer and a functional material layer to define the plasmonic nanocavities (fig. S3E). Then, an insulating PMMA layer with a thickness of ~80 nm was spin-coated onto the sample (fig. S3F), which was subsequently baked at 120°C for 3 min and partially etched by O_2_ plasma to expose the top of the gold nanocubes (fig. S3G). Last, a 50-nm–thick ITO layer (working as the top electrode) was sputtered on the top through a soft PDMS shadow mask, used to define its shape and position (fig. S3H).

### TEM characterization

For cross-sectional TEM characterization of electrically driven plasmonic nanocavities, an electron-transparent cross-sectional lamella of a selected nanocavity was prepared as follows. First, electrically driven plasmonic nanocavities were fabricated on a silicon substrate using the approach introduced above. Second, a layer of carbon (~150 nm in thickness) was sputtered on the sample to protect the nanocavities. Then, an electrically driven plasmonic nanocavity was selected under scanning electron microscopy, and a cross-sectional lamella of the nanocavity with a thickness of ~100 nm was obtained using a focused ion beam system (Helios G4, Thermo Fisher Scientific). Last, the lamella was transferred onto a copper grid and imaged using a high-resolution transmission electron microscope (F200X G2, Talos) operated at 200 kV.

### Numerical simulation

Numerical simulations of electrically driven plasmonic nanocavities were performed using a finite element method (COMSOL Multiphysics software). The geometry of the electrically driven plasmonic nanocavity used for the simulations, schematically shown in fig. S7A, was set to match that of the experimentally measured structures (derived from cross-sectional TEM images). The refractive index of the single-crystal gold was taken from Olmon *et al.* (*57*), while the refractive index of ITO was taken from the experimentally measured data (as shown in fig. S8). The refractive indices of silica, alumina, CTAB, and PMMA were set to be 1.45, 1.70, 1.44, and 1.48, respectively. The permittivity of the semiconductor monolayer is anisotropic with the in-plane components including the contributions from the excitons, modeled via Lorentz oscillators

$$\varepsilon_{\text{in}-\text{plane}}\left(\omega \right)=\varepsilon_{\infty }-f\frac{\omega_{e}^{2}}{\omega^{2}-\omega_{e}^{2}+i\omega \kappa }$$ (1)

where $\varepsilon_{\infty }$ is the high frequency component of the permittivity, while $\omega_{e}$ and κ are the frequency and linewidth of the exciton, respectively, f is the reduced oscillator strength of the excitons related to their number. For WSe_2_, the following parameters were used: $\varepsilon_{\infty }=17.5$, $\hbar \omega_{e}=1.67\;\text{eV}$, and κ=60meV; and for WS_2_, $\varepsilon_{\infty }=18$, $\hbar \omega_{e}=1.96\;\text{eV}$, and κ=95meV. The out-plane component of the permittivity was set to be 7.4 for WSe_2_ and 5.9 for WS_2_ (*58*).

The dark-field scattering spectra were modeled in the scattering formulation. The nanocavity was illuminated with a transverse electric (TE)-polarized electromagnetic plane wave at 80° to match the experimental conditions. The wavelength of the incident wave was varied from 500 to 900 nm, while the power flow of the scattered fields was integrated inside a 64° collection angle corresponding to the numerical aperture (NA) of the objective used in the experiments, 500 nm from the center of the nanocube bottom face. The normalized electric near-field distributions ($|\;E\;|/|\;E_{0}\;|$) and the surface charge density distributions (ρ) for various modes were obtained from the full vector field given by the numerical solution. Eigenmode simulations were performed using a built-in eigenfrequency solver.

### Analytical calculation of mode dispersion

The process of coupling of *N* quantum emitters with an optical mode is described by Tavis-Cummings theory using the following Hamiltonian (*59*)

$$\begin{array}{c} H=\sum_{i}E_{e}{\hat{\alpha }}_{i}^{†}{\hat{\alpha }}_{i}+E_{\text{pl}}{\hat{a}}^{†}\hat{a}+\hbar \sum_{i}g_{0}\left({\hat{\alpha }}_{i}{\hat{a}}^{†}+{\hat{\alpha }}_{i}^{†}\hat{a}\right) \end{array}$$ (2)

where, in our case, ${\hat{\alpha }}_{i}$(${\hat{\alpha }}_{i}^{†}$) is the annihilation (creation) operator for the excitons and $\hat{a}$(${\hat{a}}^{†}$) are the corresponding operators for the plasmonic mode, while $g_{0}$ is the coupling strength between a single exciton and the plasmonic mode. Solving the eigenmode problem, one obtains *N* − 1 dark states and two optically active states, related to the hybridization of the plasmonic mode with a superoscillator produced by a coherent ensemble of the excitons (*29*, *32*). The energies of the hybridized states are given by the following

E±=12(Epl+Ee)±g2+14(Epl−Ee)2 (3)

where $E_{e}$ and $E_{\text{pl}}$ are the energies of the excitons and the quanta of the plasmonic mode and $g=\sqrt{N}\cdot g_{0}$ is the coupling strength of the plasmonic mode and the excitonic superoscillator. This gives the following expression for the frequencies of the hybridized modes

$$\begin{array}{c} \omega_{\pm }=\frac{1}{2}\left(\omega_{e}+\omega_{\text{pl}}\right)\pm \sqrt{g^{2}+\frac{1}{4}\delta^{2}} \end{array}$$ (4)

where $\delta =\omega_{\text{pl}}-\omega_{e}$ presents the detuning between the frequencies of the plasmonic mode and excitons.

### Estimation of number of excitons involved in strong coupling

For the system that has *N* excitons coupled with a single plasmonic mode, the total Rabi splitting can be approximately calculated as follows (*12*)

ΩR=μm4πℏNcλεε0V (5)

where λ and $\mu_{m}=7.675$ D are the transition wavelength and the transition dipole moment of excitons in a WSe_2_ monolayer (*58*), respectively, ε is the permittivity of the medium surrounding the excitonic material, ℏ is the reduced Planck constant, and $\varepsilon_{0}$ is the free-space permittivity. The effective mode volume of the plasmonic mode V can be estimated by integrating the energy density W(r) over the region of the nanogap first and then normalizing the result to its maximum value (*60*)

V=∫W(r)drmax[W(r)] (6)

Thus, using the experimentally measured Rabi splitting and the calculated mode volume, the number of involved excitons during the modulation process can be calculated.

### Light emission characterization setup

Upon the application of a static bias voltage from a source meter (2611B, Keithley), the light emission from individual nanocavities was collected by a 100× objective (NA = 0.9; TU Plan Fluor, Nikon) and directed through a beam splitter to a charge-coupled device (CCD) camera for imaging and to a spectrometer [combination of a monochromator (Kymera 193i, Andor) and an electron-multiplying CCD (EMCCD) camera (iXon Ultra, Andor)] for the spectral analysis, as schematically shown in fig. S20A. A color CCD camera (DS-Fi3, Nikon) was used for imaging of excitonic luminescence from a semiconductor monolayer, while an EMCCD camera (iXon Ultra, Andor) was used for the measurement of inelastic tunneling–induced light emission. Then, the emission spectrum P(λ) was obtained by correcting the measured emission spectrum $P_{\text{meas}}\left(\lambda \right)$ with a spectral response of the measurement setup, T(λ), which includes the wavelength-dependent transfer efficiency of all optical elements in the microscope and detection efficiency of the spectrometer. The obtained transfer function T(λ) is presented in fig. S20B.

### Estimation of EQE for excitonic luminescence

In the case of excitonic luminescence from an electrically interfaced plasmonic nanocavity functionalized with a WS_2_ monolayer, the EQE can be calculated as a ratio between the number of the emitted photons and surface plasmon polaritons propagating along interface between the gold flake and PMMA, $N_{\text{opt}}$, and the number of the tunneled electrons, $N_{e}$, in a given period of time

EQE=Nopt/Ne (7)

To estimate $N_{\text{opt}}$, the light emission power from individual nanocavities, $P_{\text{meas}}$, was first measured using an objective with NA = 0.9 and then corrected to obtain the emitted power in all directions, $P_{\text{opt}}$, using a transfer function, $T\left(\lambda_{0}\right)$ (where $\lambda_{0}$ is the wavelength of the emitted photons; fig. S20B) and a simulated angular emission characteristics of the plasmonic mode (see below). The number of the emitted photons and plasmons, $N_{\text{opt}}$, was calculated by dividing the corrected total emitted power, $P_{\text{opt}}$, by the photon energy [for simplicity, all of the emitted photons were assumed to have the same wavelength of $\lambda_{0}$ = 620 nm for the case of the forward bias (Fig. 5C) and $\lambda_{0}$ = 640 nm for the case of the backward bias (Fig. 5E)]. The number of the tunneled electrons can be extracted from the tunneling current dividing it by the electron charge.

The numerical simulations of the angular emission characteristics were performed using a finite element method (COMSOL Multiphysics software) using a model schematically shown in fig. S7B. A dipole source was put in the middle of the dielectric gap to mimic the exciton emission source in the semiconductor monolayer, and its wavelength was set be 620 nm for neutral excitons and 640 nm for charged excitons. In the experiment, excitons can be generated everywhere in the semiconductor layer. Therefore, the ratio, $\eta_{\text{opt}}$, between emission power flow, $P_{\text{rad}}$, integrated inside a 64° collection angle corresponding to the NA of the objective and total power flow, $P_{\text{tot}}$, integrated over the upper semisphere 500 nm away from the bottom surface of the cube ($\eta_{\text{opt}}=P_{\text{rad}}/P_{\text{tot}}$) was obtained by modelling various dipole positions uniformly scanning the nanogap area (square grid, step 5 nm), and then the results were averaged to obtain the average ratio, $\eta_{\text{opt}\_\text{ave}}$. The corrected total emitted power, $P_{\text{opt}}$, can be obtained by dividing the measured emitted power, $P_{\text{meas}}$, by the transfer function, $T\left(\lambda_{0}\right)$, and the simulated average ratio, $\eta_{\text{opt}\_\text{ave}}$.

## Supplementary Material

20250528-1

## Supplementary Materials

### This PDF file includes:

Figs. S1 to S25

Correction (20 March 2026):
In the originally published versions of Fig. 2, fig. S11, and fig. S12, the scale bars were incorrectly labeled. In Fig. 2, E and F, the scale bar labels have been corrected from 25 μm to 25 nm. In fig. S11, B to E, and fig. S12, B and C, the scale bar labels have been corrected from 20 μm to 20 nm. The Supplementary Materials and online and PDF versions of the article have been updated. The original version of the Supplementary Materials is available here:
