# Supplementary material for: Active control of excitonic strong coupling and electroluminescence in electrically driven plasmonic nanocavities
Source: Sci Adv. 2025 May 28;11(22):eadt9808. doi: 10.1126/sciadv.adt9808 (PMC12118593; doi:10.1126/sciadv.adt9808)
Supplement: Supplementary file 3 — Correction (20 March 2026): In the originally published versions of Fig. 2, fig. S11, and fig. S12, the scale bars were incorrectly labeled. In Fig. 2, E and F, the scale bar labels have been corrected from 25 μm to 25 nm. In fig. S11, B to E, and fig. S12, B and C, the scale bar labels have been corrected from 20 μm to 20 nm. The Supplementary Materials and online and PDF versions of the article have been updated. The original version of the Supplementary Materials is available here: [file sciadv.adt9808_sm.v1.pdf]

Science Advances
AAAS

Supplementary Materials for

Active control of excitonic strong coupling and electroluminescence in electrically driven plasmonic nanocavities

Junsheng Zheng *et al.*

Corresponding author: Anatoly V. Zayats, a.zayats@kcl.ac.uk; Limin Tong, phytong@zju.edu.cn; Pan Wang, nanopan@zju.edu.cn

Sci. Adv. **11**, eadt9808 (2025)
DOI: 10.1126/sciadv.adt9808

This PDF file includes:

Figs. S1 to S25

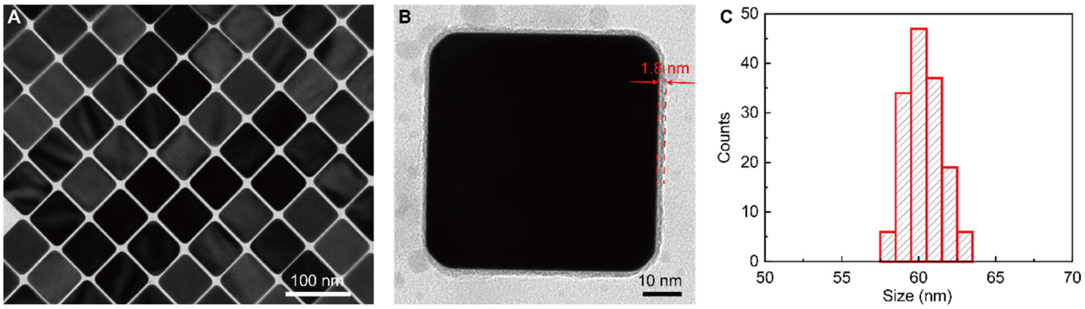

Fig. S1.

Characterization of gold nanocubes. (**A**) Transmission electron microscopy (TEM) image of gold nanocubes with an average size of 60 nm. (**B**) TEM image of a gold nanocube, which shows a bilayer of cetyltrimethylammonium bromide (CTAB) with a thickness of ~1.8 nm on the nanocube surface. (**C**) Size distribution of 150 gold nanocubes.

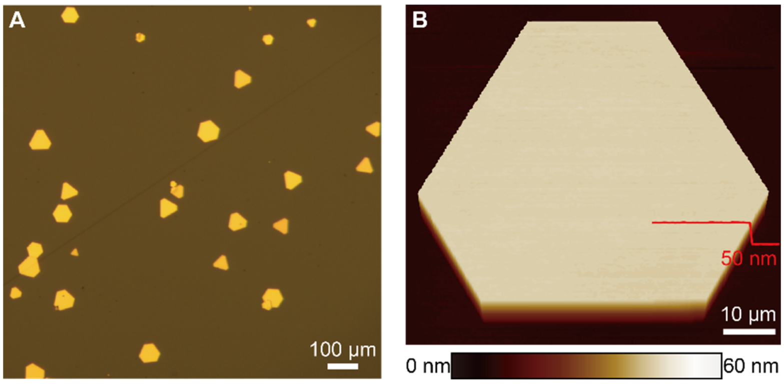

Fig. S2.

Characterization of gold flakes. (**A**) Optical micrograph of gold flakes synthesized on a glass slide taken in reflection. (**B**) Atomic force microscopy image of a 50-nm-thick gold flake, confirming its smooth surface with a root-mean-square roughness of ~0.3 nm. The red line shows the height profile near the edge of the gold flake in the specified region.

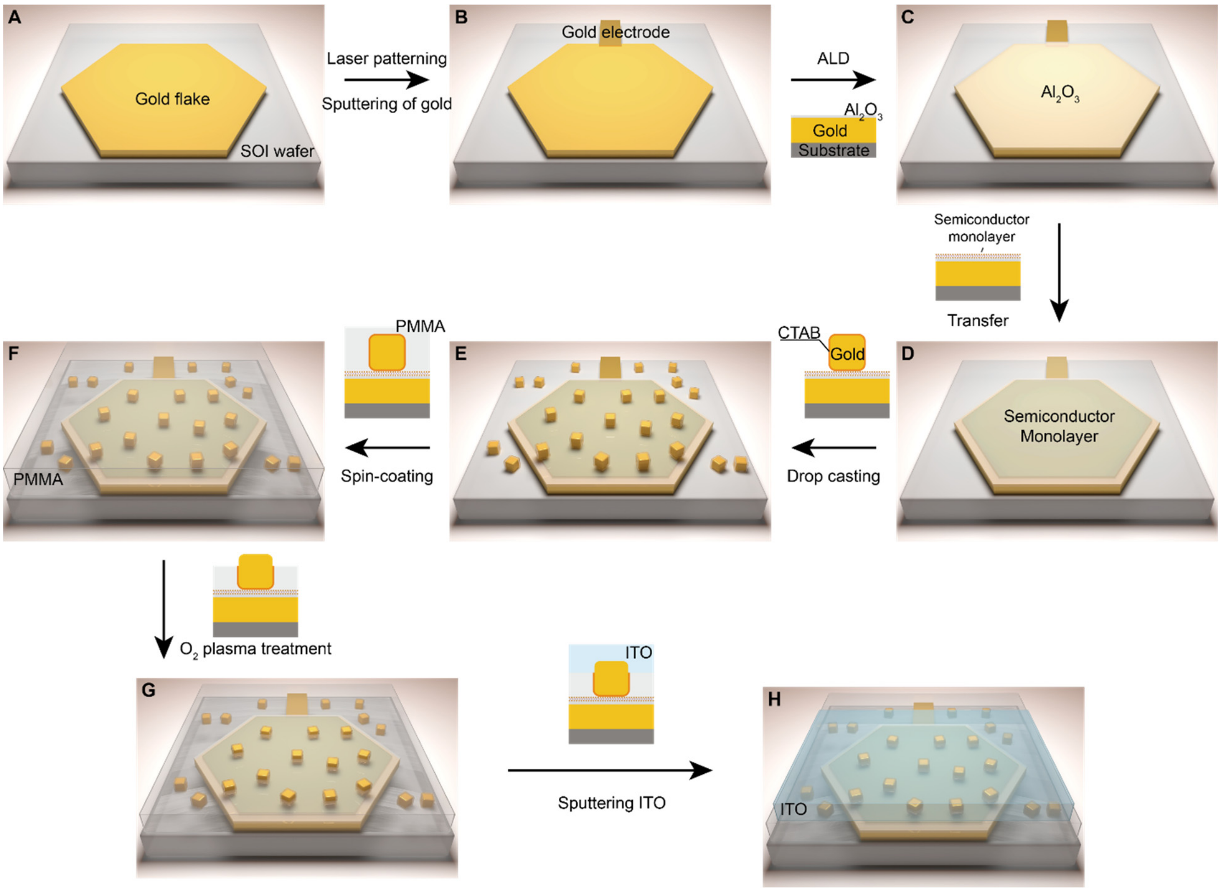

Fig. S3.

Procedure for fabrication of electrically driven functional plasmonic nanocavities. (**A**) Chemically synthesized single-crystal gold flakes with a thickness of ~50 nm is transferred onto a silicon substrate (covered with a 300-nm thick SiO_2 layer). (**B**) A gold strip electrode (20 μm in width) is fabricated at the edge of the gold flake for electric connection, using direct laser writing, followed by deposition of a Cr/Au (5/50 nm) layers and lift-off. (**C**) An alumina spacer is deposited using molecular-assisted atomic layer deposition (ALD). (**D**) A monolayer of WSe_2 or WS_2 is transfer printed onto the top of the alumina layer (see fig. S4 for transfer printing details). (**E**) Gold nanocubes are drop-casted to form functional plasmonic nanocavities. (**F**) An insulating poly(methyl methacrylate) (PMMA) layer is spin-coated onto the sample, and subsequently baked at 120 °C for 3 min. (**G**) The PMMA layer is partially etched by O_2 plasma to expose the top of the gold nanocubes. (**H**) A 50-nm-thick indium tin oxide (ITO) layer is deposited to form the top electrode.

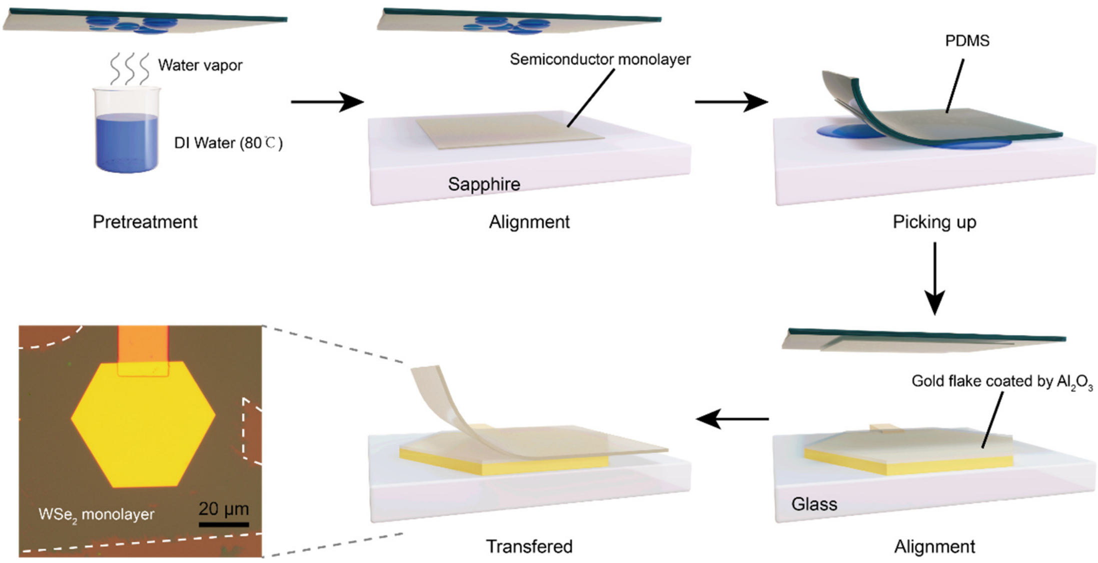

Fig. S4.

Schematic diagram of PDMS-assisted transfer printing of a semiconductor monolayer onto targeted gold flake. Firstly, a small patch of PDMS used for transfer printing of a semiconductor monolayer was treated with water vapor to form numerous micrometer-scale water droplets on it. Secondly, under an optical microscope, the PDMS was quickly aligned (usually within a minute) and softly pressed onto the targeted semiconductor monolayer, then it was slowly withdrawn backward to pick it up. Then, the monolayer attached to the PDMS film was aligned and brought into a full contact with the targeted region. Finally, the semiconductor monolayer was left on the gold flake after a careful withdrawal of the PDMS film. Inset, optical micrograph of a gold flake (coated with a 3.1-nm-thick alumina spacer) after the transfer printing of a WSe_2 monolayer (outlined by white dashed lines) onto it.

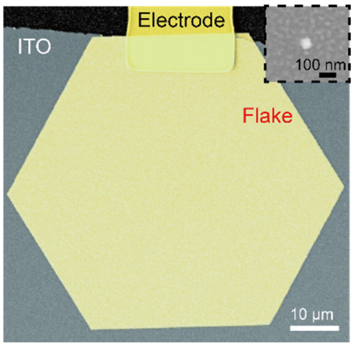

Fig. S5.

Characterization of electrically driven bare plasmonic nanocavity. False-color scanning electron microscopy (SEM) image of an as-fabricated structure with many electrically driven plasmonic nanocavities formed on a gold flake. Inset, enlarged SEM image of an individual electrically driven bare nanocavity formed on the flake.

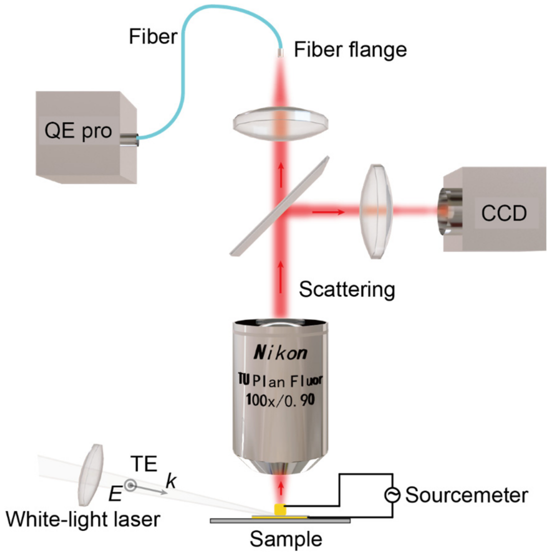

Fig. S6.

Dark-field spectroscopy setup. Schematic diagram of the dark-field setup for light scattering imaging and spectroscopy of electrically driven plasmonic nanocavities. Briefly, a TE-polarized beam from a white-light laser (FIU-6, NKT Photonics) was first focused onto nanocavities at an oblique angle of about 80° with respect to the surface normal. The scattering signal from individual nanocavities was first collected by a 100× objective (NA = 0.9, TU Plan Fluor, Nikon), and then directed with a beam splitter to a charge-coupled device (CCD) camera (DS-Fi3, Nikon) for imaging and a spectrometer (QE Pro, Ocean Optics) for spectral analysis. All the measured scattering spectra were calibrated by the spectrum of the white-light laser and spectral response of the detection system.

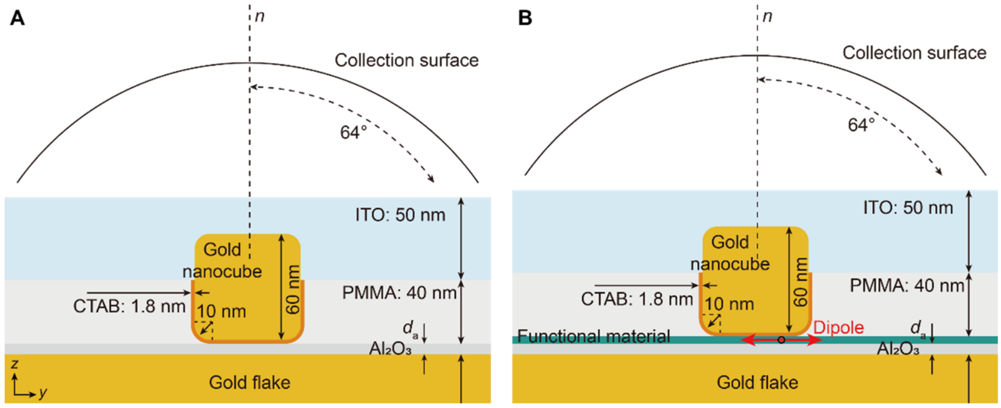

Fig. S7.

Numerical simulation models of plasmonic nanocavities. (**A** and **B**) Schematic illustrations of models used for (A) numerical simulations of scattering spectra, electric near-field and surface charge density distributions and (B) excitonic luminescence from a WS_2 monolayer.

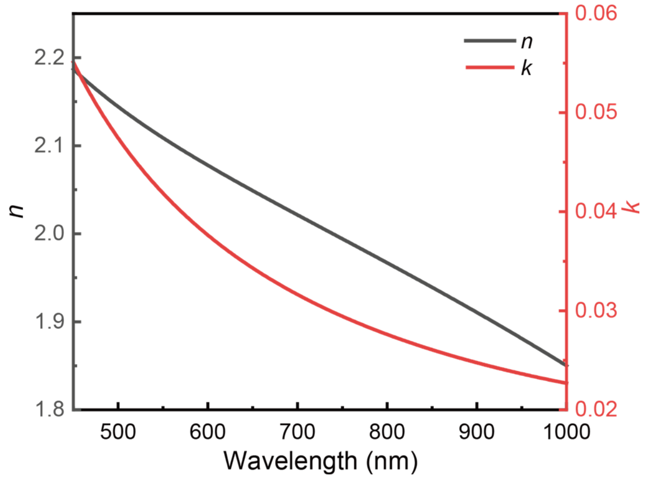

Fig. S8.

Refractive index of ITO electrode. Measured real (black line) and imaginary (red line) parts of the refractive index of a 50-nm-thick ITO layer deposited by sputtering in a mixed gas atmosphere (98 sccm Ar and 2 sccm O_2) with DC power of 350 W for 180 s at room temperature.

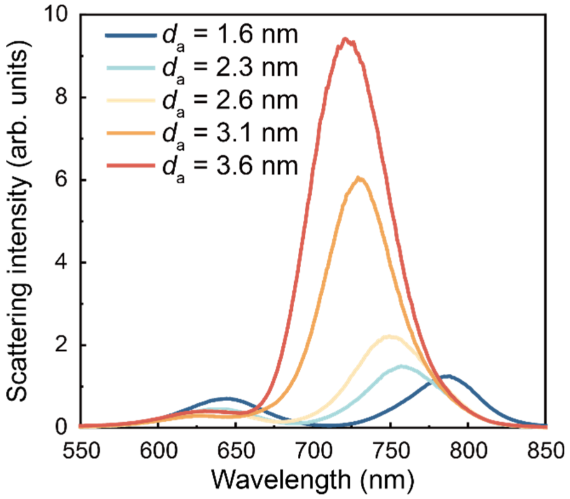

Fig. S9.

Effect of alumina thickness on nanocavity optical properties. Experimentally measured scattering spectra of electrically driven bare plasmonic nanocavities with varying thickness of the alumina spacer.

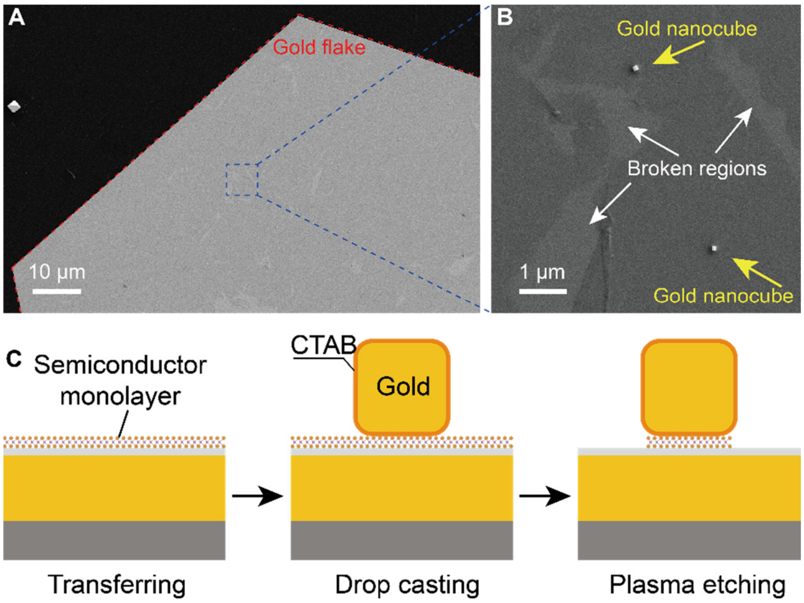

Fig. S10.

Characterization of WSe_2-functionalized nanocube-on-mirror plasmonic nanocavity and procedure for removing semiconductor monolayer outside nanocavities via oxygen plasma etching. (**A**) Scanning electron microscope image of a gold flake after the depostion of 3.1-nm-thick alumina, a monolayer of WSe_2 and gold nanocubes. The red dashed line indicates the outline of the gold flake, which is fully covered by the WSe_2 monolayer. (**B**) Enlarged view of the region marked by a dashed blue rectangle in (A), in which the WSe_2 monolayer and two gold nanocubes can be clearly seen. (**C**) After the transfer printing of the semiconductor monolayer on to the gold flake coated with an alumina spacer, gold nanocubes are drop-casted to form plasmonic nanocavities. The monolayer outside the nanocavities is then removed by an oxygen plasma etching process to eliminate its influence on the measurement of photoluminescence (PL).

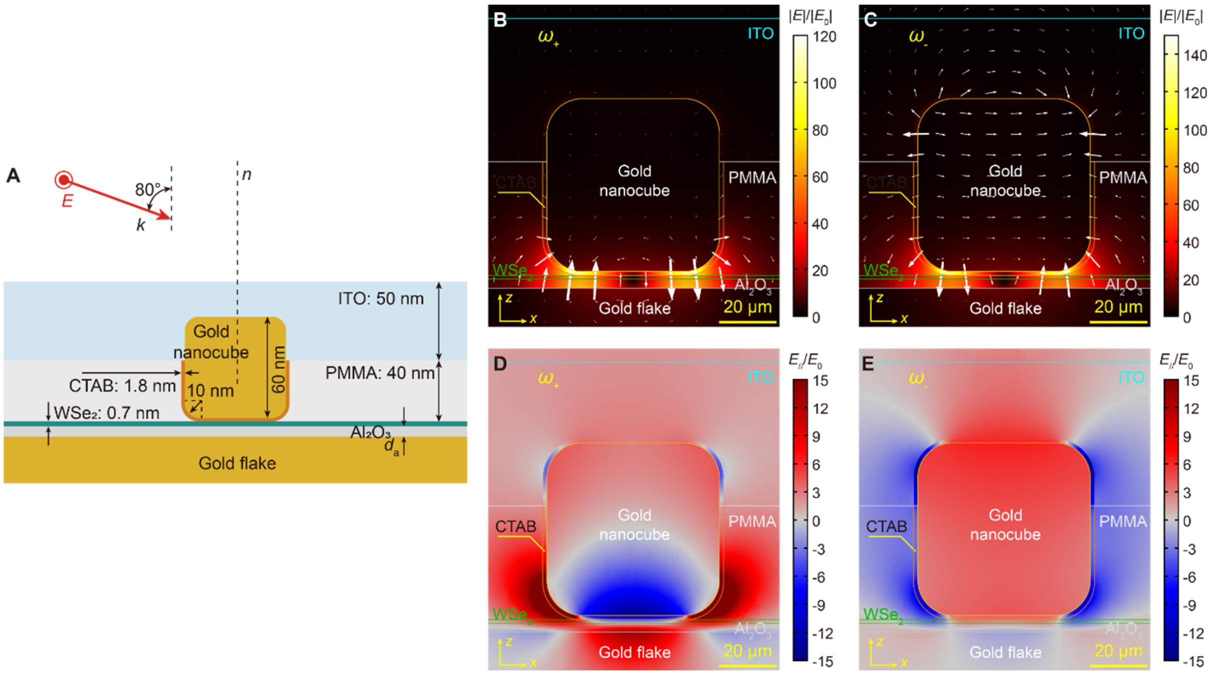

Fig. S11.

Numerical simulated electric field maps of hybridized states in WSe_2-functionalized electrically driven plasmonic nanocavities using a scattering formulation. (**A**) Schematic illustration of the model used for numerical simulation of electric field maps. (**B** and **C**) Maps of electric field enhancement for (B) the upper and (C) the lower polariton resonances, complemented with the corresponding vectorial distributions of the electric field. (**D** and **E**) Maps of the enhancement of the in-plane component of the electric field for (D) the upper and (E) the lower polariton resonances.

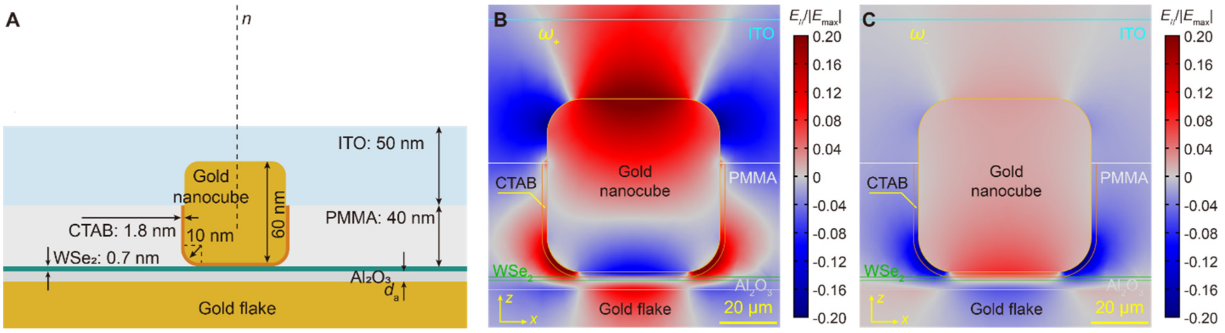

Fig. S12.

Numerical simulated eigenmode maps of hybridized states in WSe_2-functionalized electrically driven plasmonic nanocavities using a eigenfrequency solver. (**A**) Schematic illustration of the model used for eigenmode numerical simulations with indicated model parameters. (**B** and **C**) Nunerically simulated distributions of the in-plane component of the electric field for the eigenmodes of WSe_2-functionalized plasmonic nanocavity presenting (B) the upper (ω_+) and (C) the lower (ω_-) plasmon-exciton resonances. The magnitude of the in-plane electric field component is normalized to the global maximum of the mode electric field.

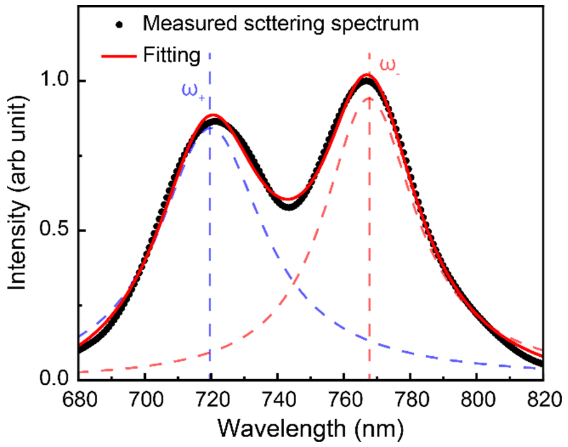

Fig. S13.

An example for extracting polaritonic energies from a typical scattering spectrum. An example for extracting polaritonic energies from a typical scattering spectrum of a WSe_2-functionalized electrically driven plasmonic nanocavities, using fitting the spectrum with two Lorentzian line shapes corresponding to the hybridized polaritonic modes.

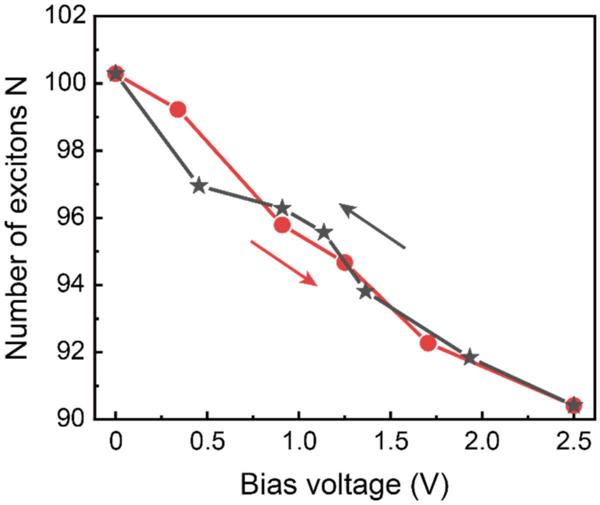

Fig. S14.

Number of excitons involved in modulation of strong coupling. Estimated number of excitons involved in the modulation of strong coupling effect presented in Fig. 4A, which decreases from ~100 to ~90 with the increase of the bias voltage from 0 to 2.5 V, and then increases back to ~100 with the decrease of the bias voltage back to 0 V.

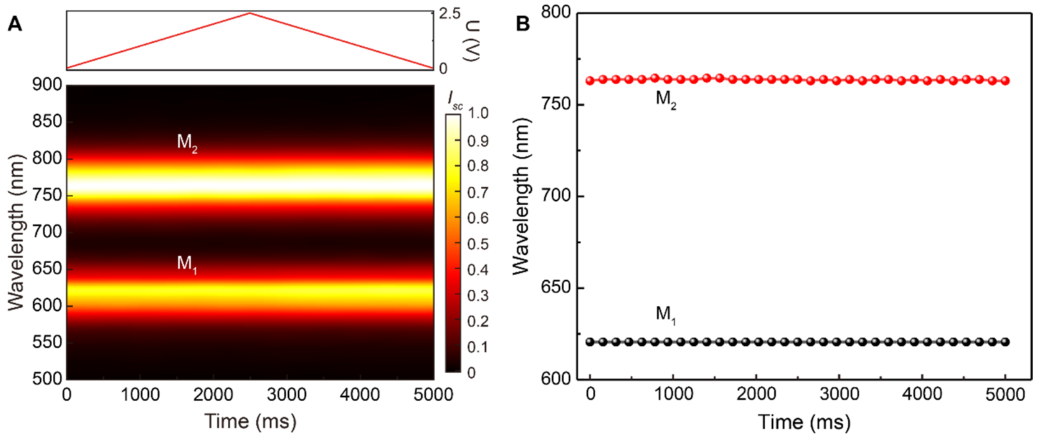

Fig. S15.

Investigation of the effect of strong electric field on optical response of electrically driven bare plasmonic nanocavity (d_a = 3.1 nm). (**A**) Spectral map showing the evolution of the scattering spectra of an electrically driven bare plasmonic nanocavity under a continuously increasing and then a reversely decreasing bias as indicated in the upper panel. The color represents the normalized scattering intensity. (**B**) Time dependence of the peak wavelengths for mode M_1 (black dotted line) and M_2 (red dotted line) extracted from (A).

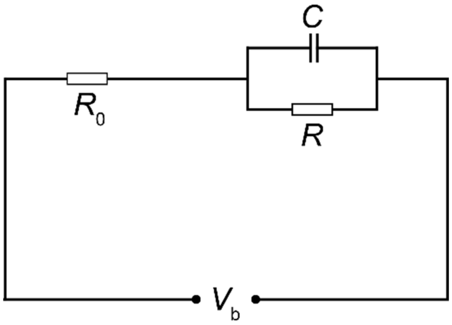

Fig. S16.

Equivalent circuit of the strong-coupling modulation device. In the equivalent circuit, R and C are the resistance and capacitance of the electrically driven plasmonic nanocavities, R_0 is the resistance of the rest of the circuit (including the resistances of the gold electrodes, ITO layer and alumina wire used for wire bonding in the circuit), which is estimated to be ~220 ohm. The capacitance is numerically calculated to be $\sim 1.15\times10^{-15}$ F for the device containing ~50 nanocavities with the same structural parameters. For the strong-coupling modulation device operating under a bias of 2.5 V, the tunnelling current going through the device is measured to be around 1.5 nA, which gives R ~1.7 Gohm. Therefore, the RC time constant of the system is $\tau = R_0R/(R_0+R)\cdot C \approx R_0C = 242$ fs.

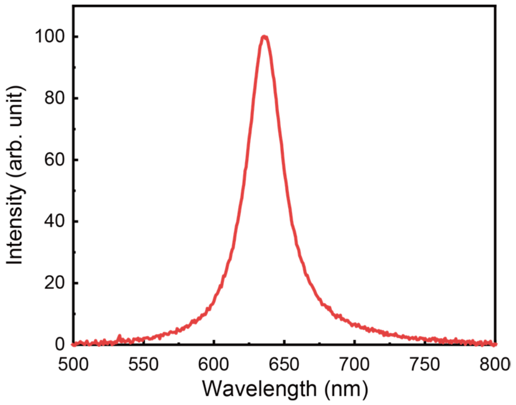

Fig. S17.

Photoluminescence spectrum of a WS_2 monolayer. Photoluminescence spectrum of a WS_2 monolayer measured on a sapphire substrate under a 532 nm excitation at room temperature, which has a relatively broad linewidth of 31 nm. The spectrum is dominated by negatively charged excitons, but also has some contributions from neutral excitons, bound excitons and biexcitons, which leads to its broadening.

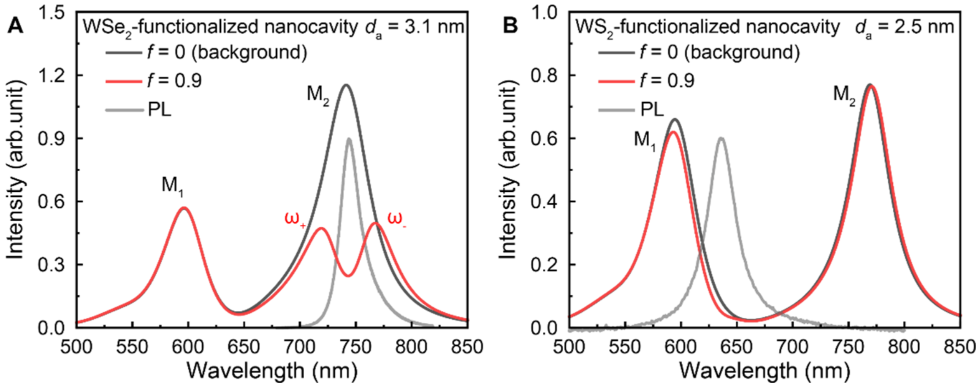

Fig. S18.

Numerical simulations of nanocavity coupling states depending on the different gap thickness and functional materials. (**A** and **B**) Numerically simulated dark-field scattering spectra (red lines, $f = 0.9$: excitons are present) when the excitonic resonance of a semiconductor monolayer (A) matches (WSe_2, $d_a = 3.1$ nm, first set of experiments), and (B) does not match (WS_2, $d_a = 2.5$ nm, second set of experiments) a plasmonic mode of electrically driven plasmonic nanocavities (black lines, $f = 0$: excitons are not present). Factor f represents the oscillator strength of the excitons used in the simulations (for details see Materials and Methods). For reference, the PL spectra (gray lines) from (A) a WSe_2 and (B) a WS_2 monolayer on a sapphire substrate are provided.

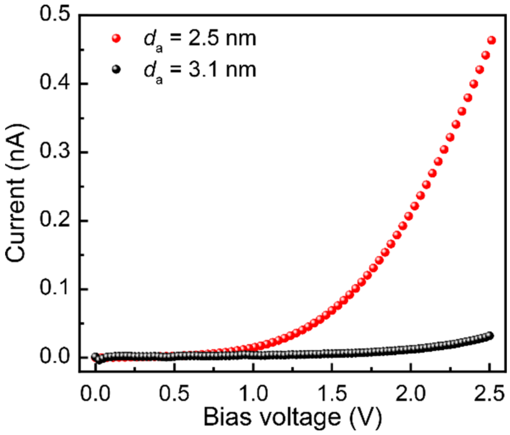

Fig. S19.

Alumina thickness induced variation on tunnelling current. Experimentally measured tunneling current from single WSe_2-functionalized electrically driven plasmonic nanocavities with an alumina thickness of 3.1 nm (black circles) and WS_2-functionalized nanocavities with an alumina thickness of 2.5 nm (red circles). The current is obtained by dividing the measured total tunnelling current through the entire device by the total number of the nanocavities in it.

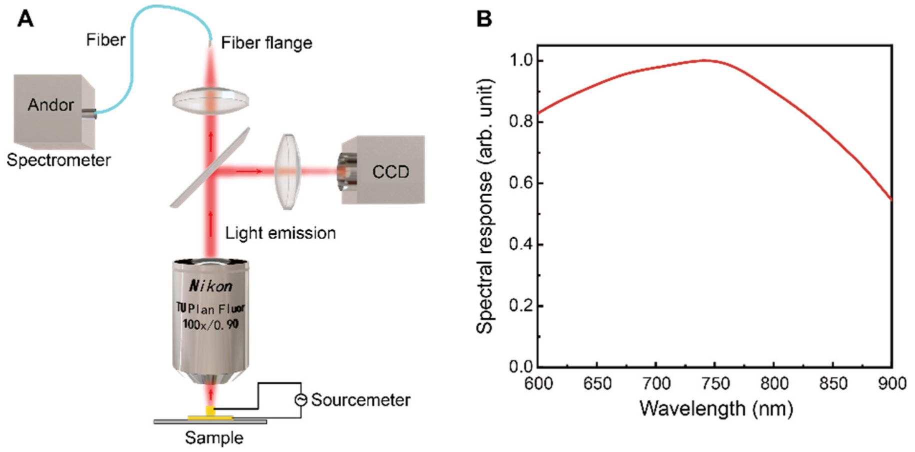

Fig. S20.

Light emission characterization setup. (**A**) Schematic diagram of the setup for imaging and spectroscopy of light emission from electrically driven plasmonic nanocavities. (**B**) Spectral response of the measurement setup, which includes the spectral response of all optical elements in the microscope and detection efficiency of the spectrometer.

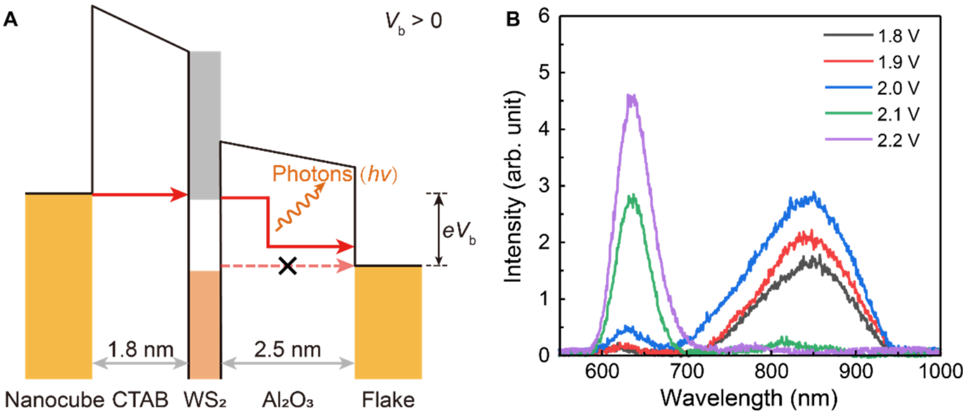

Fig. S21.

Electroluminescence (EL) spectra of electrically driven plasmonic nanocavities functionalized with a WS_2 monolayer under varied forward biases. (**A**) Schematical illustration of a process of IET-assisted light emission. (**B**) Bias-dependent light emission spectra measured from an electrically driven plasmonic nanocavity functionalized with a WS_2 monolayer, which shows a clear transition from inelastic tunnelling excited luminescence to excitonic EL with the increase of the applied forward from 1.8 to 2.2 V. In the case of backward bias, a separate IET-assisted EL was not observed due to the much lower tunnelling current.

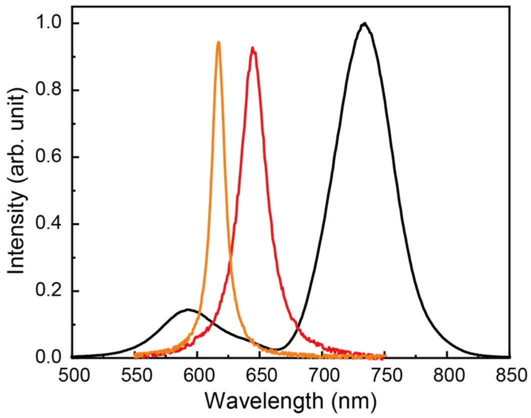

Fig. S22.

Scattering and EL spectra of electrically driven plasmonic nanocavities functionalized with a WS_2 monolayer. Normalized scattering spectrum (black line) of the plasmonic nanocavity having a alumina thickness of 2.5 nm and functionalized with a monolayer WS_2 (marked in the inset of Fig. 3b). The emission spectra from the nanocavity under 2.3 V (red line) and -3.8 V (orange line) biases are shown for comparison (the same curves from Figs. 5C and 5E).

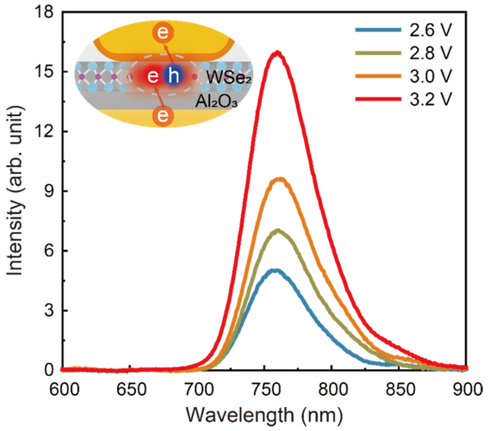

Fig. S23.

EL spectra from a WSe_2 monolayer. Bias-dependent excitonic EL spectra measured from an electrically driven plasmonic nanocavity having a alumina thickness of 2.5 nm and functionalized with a WSe_2 monolayer (schematically shown in the inset), which shows an excitonic emission peak at 754 nm with a linewidth of ~59 nm, agreeing well with the photoluminescence characteristics of negatively-charged excitons.

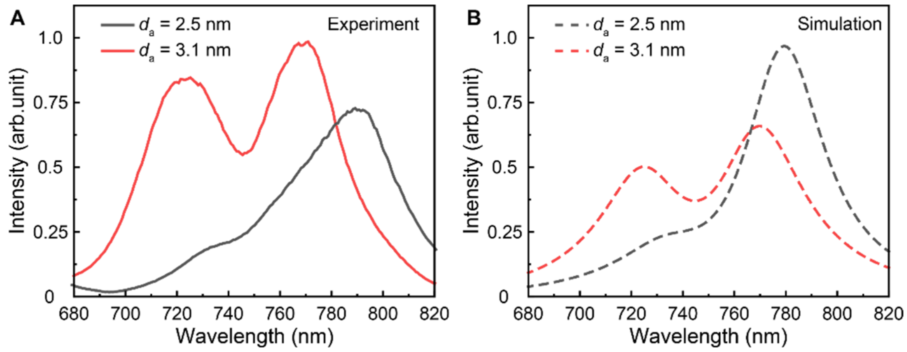

Fig. S24.

Variation of scattering spectrum with change of alumina thickness. (**A** and **B**) Measured (A) and calculated (B) scattering spectra of WSe_2 monolayer-functionalized electrically driven plasmonic nanocavities with an alumina thickness of 3.1 nm (red lines) and 2.5 nm (black lines). The oscillator strength (*f*) is set as 0.9 to present excitons for both cases (see Materials and Methods). By decreasing the alumina thickness from 3.1 to 2.5 nm, the plasmonic mode red shifts and goes out of resonance with the WSe_2 excitons, thus the system enters a weak coupling regime.

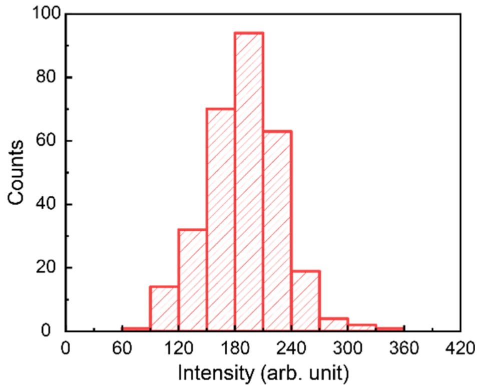

Fig. S25.

Statistical analysis of the EL uniformity. Statistical analysis of the EL intensities of WS_2-functionalized electrically driven plasmonic nanocavities under a constant bias of 2.3 V.
